# Epidemiological and clinical analyses of corneal transplants performed in a reference eye center in Recife, Brazil

**DOI:** 10.5935/0004-2749.20220074

**Published:** 2025-08-22

**Authors:** Edilana Sá Ribeiro, Anne Elizabeth Ferraz de Andrada, Taciana Mirely Maciel Higino, Isadora Diógenes Lopes, Adriano Cabral de Vasconcelos, Analine Lins de Medeiros, Camilla Silva da Rocha, Camila V. Ventura

**Affiliations:** 1 Department of Ophthalmology, Fundação Altino Ventura, Recife, PE, Brazil; 2 Department of Ophthalmology, Hospital de Olhos de Pernambuco, Recife, PE, Brazil; 3 Department of Research, Fundação Altino Ventura, Recife, PE, Brazil

**Keywords:** Corneal disease/epidemiology, Corneal transplantation, Keratoplasty, penetrating, Brazil/epidemiology, Doença da córnea/epidemiologia, Transplante de córnea, Ceratoplastia penetrante, Brasil/epidemiologia

## Abstract

**Purpose:**

To evaluate the epidemiological and clinical profiles of corneal transplants performed in a reference eye center in Recife, state of Pernambuco, Northeastern Brazil.

**Methods:**

This cross-sectional study collected epidemiological and clinical data from the medical records of patients who underwent keratoplasty at the Altino Ventura Foundation between January and December 2017.

**Results:**

A total of 356 procedures were performed in 327 patients, of whom 165 (50.5%) were female. The mean age at surgery was 50.9 ± 22.6 years (range, 10-89 years). Most patients (n=152 [46.5%]) were from the capital and metropolitan areas. The mean waiting time for keratoplasty was 52.4 ± 58.9 days (range, 0-460 days). The main indications for keratoplasty were infectious keratitis (n=88 [24.7%]), keratoconus (n=80 [22.5%]), and previous transplant failure (n=75 [21.1%]). Penetrating keratoplasty was the most common surgical technique performed (n=213 [59.9%]) and more frequently performed in men (n=132 [76.7%]), whereas posterior lamellar transplant (n=143 [41.1%]) was more frequently performed in women (p<0.001).

**Conclusion:**

Infectious keratitis was the main indication for keratoplasty, which was similarly performed in economically active adults of both sexes. Penetrating keratoplasty was more frequently performed in men and lamellar transplants in women.

## Abstract (pt)

**Objetivo:**

Avaliar o perfil clínico e epidemiológico dos transplantes de córnea realizados em um centro de referência oftalmológica de Recife no estado de Pernambuco, localizado no nordeste do Brasil.

**Métodos:**

Esse estudo transversal coletou através de prontuários médicos dados clínicos e epidemiológicos de pacientes submetidos a ceratoplastia na Fundação Altino Ventura, de janeiro a dezembro de 2017.

**Resultados:**

Um total de 356 procedimentos foram realizados em 327 pacientes dos quais 165 (50.5%) eram mulheres. A média de idade na cirurgia foi de 50.9 ± 22.6 anos (variação, 10 - 89 anos). A maioria dos pacientes (n=152 [46.5%]) era da capital e região metropolitana. A média de tempo de espera na fila para o transplante de córnea foi de 52.4 ± 58.9 dias (variação, 0 - 460 dias). As principais indicações de transplante foram ceratite infecciosa (n=88 [24.7%]), ceratocone (n=80 [22.5%]) e falência de transplante prévio (n=75 [21.1%]). Transplante penetrante foi a técnica mais realizada (n=213 [59.9%]) e foi mais comum em homens (n=132 [76.7%]), enquanto os transplantes lamelares posteriores (n=143 [41.1%]) foram mais realizados nas mulheres (p<0.001).

**Conclusão:**

Ceratites infecciosas foram a causa mais comum de transplante, com prevalência similar em adultos economicamente ativos de ambos os sexos. Transplante penetrantes foram os prevalentes em homens e os transplantes lamelares em mulheres.

## INTRODUCTION

Corneal diseases are the second leading cause of reversible blindness in the world and confer substantial visual and psychosocial burdens on patients and impact countries economically, as it usually affects the young and active population^(1)^.

Corneal transplants or keratoplasties consists of replacement of the diseased or damaged host cornea with a healthy donor cornea^(2)^. Considered immunologically privileged owing to their physiological characteristics and avascularity, corneal transplants have a low graft rejection rate and, consequently, a high rate of success^(3)^.

With the technological advances, safety procedures, proper evaluation and storage of tissue donation, and use of anti-inflammatory and immunosuppressant drugs, better visual outcomes and quality of live have been obtained in transplanted eyes^(4,5)^. In addition, different surgical techniques have been developed to specifically address diseased or damaged corneal layers, including penetrant keratoplasties (full-thickness corneal transplants) and anterior or posterior lamellar keratoplasties (partial-thickness corneal transplants)^(6)^.

Brazil currently has the largest public organ and tissue transplant program in the world^(7-9)^. Brazilian eye banks cannot charge for the processing of donated eye tissues, regardless of the status of the institution to which the eye bank belongs (public, private, or philanthropic). This service is funded and regulated by the public health system known as *Sistema Único de Saúde*^(9)^. Among the organs transplanted in the country, the cornea is the most transplanted given the high number of donors, technical storage facility, and advanced surgical technique^(7,10)^.

In 2017, the state of Pernambuco registered an increa se of approximately 17% in corneal transplants when compared with that in the previous year and eliminated the waiting queue in the state, making it the fourth state to perform the most keratoplasties in absolute numbers in the country^(8,11)^. Of the 968 keratoplasties performed that year, the *Fundação Altino Ventura*, in Recife, the capital of the state of Pernambuco, was responsible for most cases (37.9%)^(8)^. Thus, the present study aimed to investigate the epidemiological and clinical profiles of patients who received a corneal transplant that year at Altino Ventura Foundation, a philanthropic reference eye center in Pernambuco.

## METHODS

This cross-sectional, descriptive, and analytical study evaluated the medical records of patients who underwent keratoplasty at a single center, the *Fundação Altino Ventura* (FAV), between January and December 2017. The study followed the guidelines of the Declaration of Helsinki and was approved by the institutional review board of the FAV.

The variables collected included age, sex, city of origin, corneal diagnosis, surgical waiting time, and surgical technique performed. Patients with incomplete medical records were excluded from the study.

Quantitative variables were expressed as means and standard deviations, whereas qualitative variables were presented as absolute and relative frequencies. Chi-square and Kruskal-Wallis tests were performed to assess statistical significance. Statistical tests were performed using the SPSS version 25.0 software (IBM, Chicago, USA). A p value <0.05 was considered statistically significant.

## RESULTS

Three hundred sixty-seven keratoplasties were performed in the FAV in 2017. From these cases, 11 (3.0%) had incomplete data from medical records and were excluded from the study. Three hundred fifty-six keratoplasties (343 transplants and 13 retransplants) were performed in 327 patients (165 [50.5%] males and 162 [49.5%] females). The patients’ mean age at surgery was 50.9 ± 22.6 years (range, 10-89 years), with a predominance of the 19- to 59-year age group (n=146 [44.8%]) and those from the capital and metropolitan areas (n=152 [46.5%]; Table 1).

**Table 1:** Demographic profile of the patients (n=327)

Variable	n (%)
Sex Female	165 (50.5)
Male	162 (49.5)
Age (years)	40 (12.2) 38 (11.6) 34 (10.4) 30 (9.2) 44 (13.6) 51 (15.6) 62 (19) 28 (8.6)
Origin Capital and Metropolitan areas	152 (46.5)
Countryside	151 (46.2)
Other states	24 (7.3)

Penetrant transplant was the most prevalent technique in all mesoregions, and the mean waiting time for the keratoplasty was 52.4 ± 58.9 days (range, 0-460 days).

**Figure 1:**
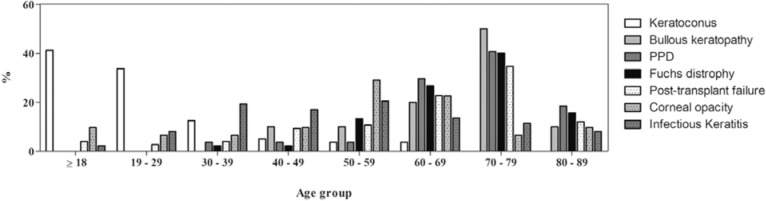
Distribution of the main diagnoses according to sex.

**Figure 2:**
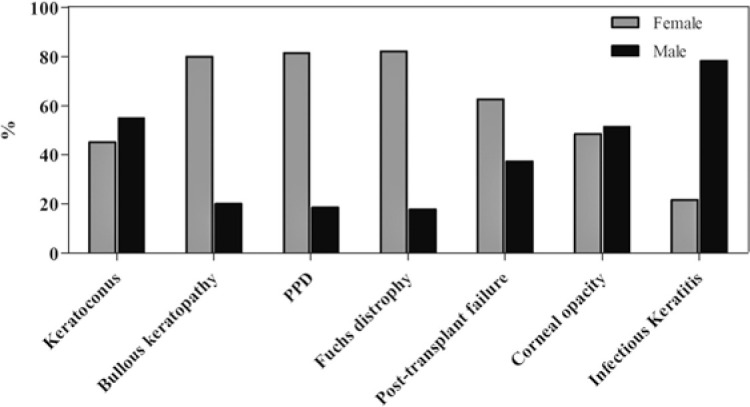
Distribution of the main diagnoses according to age range.

Keratoconus and bullous keratopathy were the main causes of transplant in the patients aged <30 and >70 years, respectively (Figure 1). Endothelial diseases were more common in the females, and infectious keratitis was more common in the males (Figure 2).

The main corneal diseases that led to corneal transplantation were infectious keratitis (n=88 [24.7%]), keratoconus (n=80 [22.5%]), and post-transplant failure (n=75 [21.1%]; Table 2). Seventy-five keratoplasties were performed owing to graft failure as follows: 31 (41.3%), primary failure; 15 (20.0%), graft rejection; and 29 (38.7%), secondary failure, of which 6 (20.6%) were from other services.

**Table 2:** Indications for corneal transplant and the surgical technique used

Variable		n (%)	
Indications for transplantation Corneal ulcer		88 (24.7)	
Keratoconus		80 (22.5)	
Post-transplant failure		75 (21.1)	
Fuchs dystrophy		45 (12.6)	
Corneal opacity		31 (8.7)	
Corneal decompensation post phacoemulsification		27 (7.6)	
Bullous keratopathy		10 (2.8)	
	Surgical techniques Penetrating transplant (n=213)		
	Optic	133 (62.4)	
	Tectonic Lamellar transplant (n=143)	80 (37.6)	
	DALK	40 (28.0)	
	DSEK	50 (35.0)	
	DMEK	53 (37.0)	

DALK= Deep anterior lamellar keratoplasty; DSEK= Descemet stripping endothelial keratoplasty; DMEK= Descemet membrane endothelial keratoplasty.

**Figure 3:**
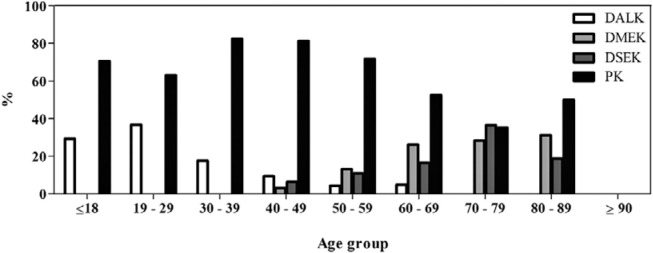
Surgical techniques used according to age (n=356). The bars represent the total number of procedures (transplants and retransplants).

**Figure 4:**
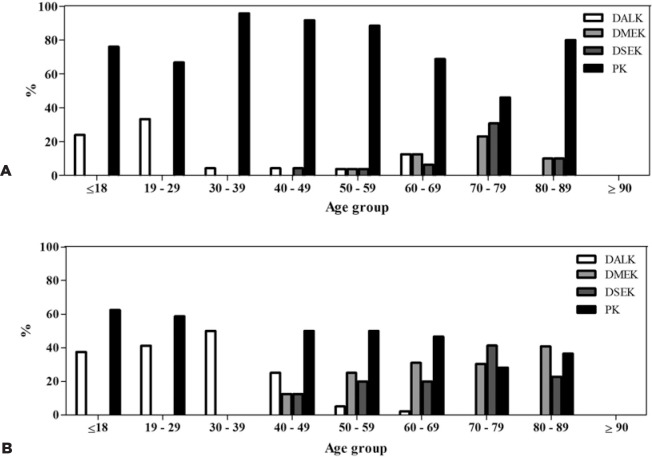
Surgical techniques used according to age group among (A) females (n=165) and (B) males (n=162). The bars represent the total number of procedures (transplants and retransplants).

With regard to the surgical technique performed, 213 patients (59.9%) underwent a penetrant keratoplasty, of whom 80 (213, 37.6%) were tectonic and 133 (213, 62.4%) were optical. Of the 143 lamellar transplants (40.1%), Descemet’s membrane endothelial keratoplasty (DMEK) was the most frequent (n=53/143 [37.0%]), followed by Descemet’s stripping endothelial keratoplasty (DSEK; n=50/143 [35.0%]) and deep anterior lamellar keratoplasty (DALK; n=40/143 [28.0%]; Table 2). The DALK technique was the most frequently performed in patients aged <50 years, while the frequencies of DMEK and DSEK increased with age (p<0.001; Figure 3). Penetrant transplantation was the most common technique performed in the males (132 [76.7%]; Figure 4A), whereas posterior lamellar transplantation prevailed in the females (103 [56.0%]; p<0.001; Figure 4B). In women aged <40 years, penetrating transplant and DALK were the most frequent, whereas in those aged >40 years, posterior lamellar keratoplasties were the most frequently performed. Among the males, penetrating transplantation was the main technique performed in all the age groups, except for the 70- to 79-year age group, for which lamellar keratoplasty was the most frequently performed (Figure 4).

DALK= Deep anterior lamellar keratoplasty; DMEK= Descemet membrane endothelial keratoplasty; DSEK= Descemet stripping endothelial keratoplasty; PK= Penetrating keratoplasty.

DALK= Deep anterior lamellar keratoplasty; DMEK= Descemet membrane endothelial keratoplasty; DSEK= Descemet stripping endothelial keratoplasty; PK= Penetrating keratoplasty.

**Figure 5:**
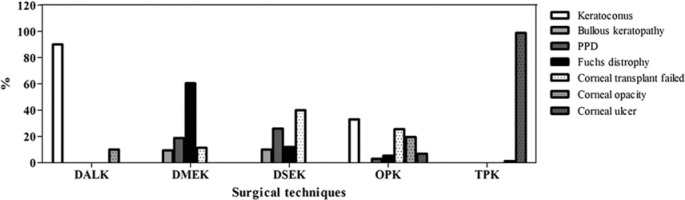
Surgical techniques used according to transplant indication. The bars represent the percentages of the procedures performed.

When the surgical technique performed was analyzed according to corneal disease, DALK and optical penetrating keratoplasty were the preferred techniques for keratoconus cases (n=36 [90.0%] and n=44 [33.1%], respectively); DMEK was preferred for Fuchs dystrophy cases (n=32 [60.4%]); DSEK was preferred for corneal transplant failure (n=26 [52.0%]), and tectonic penetrating keratoplasty was preferred for infectious keratitis (n=79 [98.8%]; p<0.001; Figure 5).

PPD= Post phacoemulsification decompensation.

## DISCUSSION

In 2017, the state of Pernambuco obtained a zero corneal transplant status, with more procedures performed, a status not achieve since 2015^(11)^. This status was maintained in subsequent years, but the numbers of transplants performed in 2018 and 2019 decreased to 775 and 812, respectively^(12,13)^. The mean waiting time for corneal transplantation in Pernambuco in 2017 was 52.4 days. In 2014, another study in Pernambuco found that 57% of patients waited 1-6 months until transplantation^(1)^. In other regions of Brazil, the waiting time for a keratoplasty can reach 3 years^(14)^. This notable reduction in waiting time in Pernambuco in 2017 was associated^(8,11)^.

One measure that could easily improve eye bank analysis would be the establishment of a national standardized data recording and analysis of data. Moreover, the waiting time for lamellar transplants could be decreased if the legislative structure that allows the use of tissues of precut anterior and posterior corneal grafts is revised^(15)^.

Although epidemiological studies have shown that keratoconus is the main indication for corneal transplants in the world, the indication may vary among continents. In Asia, infectious keratitis is the main indication, whereas in North America, bullous keratopathy is the main diagnosis that leads to corneal transplantation^(15)^. In the present study, infectious keratitis was the main indication for keratoplasty. This finding can be justified by the fact that the FAV is a public eye hospital and a reference tertiary center in the state of Pernambuco that offers 24-hour emergency department services and that many patients with infectious keratitis from other services and cities are referred to the hospital for clinical/surgical management.

Studies have shown that the incidence of infectious keratitis is related to poverty, use of traditional eye medicines, and delayed presentation for treatment, which aere common in low-income countries^(16,17)^. Therefore, this study alerts for the need of prevention and early detection measures, as they may reduce the incidence rate of corneal transplantation in developing countries.

In terms of clinical profile, the patients who underwent corneal transplantation in this study were from both sexes and the working population. Studies have estimated that the annual direct cost of blindness is approximately US$ 25 billion and the productivity losses in Brazil range from US$ 2.7 to US$ 4.5 billion per year^(18,19)^. Thus, corneal transplant in the patients in the present study generated not only a social impact but also an economic impact.

In the study sample, an association was observed between the corneal transplant technique and patient sex. A higher incidence rate of penetrating keratoplasty, especially tectonic keratoplasty, was found in men, which suggests a higher incidence rate of infectious keratitis in that population, as observed in our study. Male occupations such as agriculture and hard labor might have contributed to work-related trauma that led to keratitis^(20)^. On the other hand, posterior lamellar keratoplasties were more prevalent in women, which is related to the higher prevalence rates of females in the el derly population and Fuchs dystrophy in women, resulting in the higher incidence of endothelial diseases^(6)^.

Similar to the description of Cruz et al., evidence from the present study shows a correlation between age and surgical technique^(21)^. This relationship may be explained by the high prevalence of stromal corneal disorders such as keratoconus and infectious keratitis in younger individuals and corneal endothelial diseases in elderly patients^(6)^. In addition, ocular surgeries that can decompensate the corneal endothelium, such as phacoemulsification, are predominant in individuals with more-advanced ages^(22)^.

In addition, the present study identified 75 keratoplasties that were performed for graft failure as follows: 31 (41.3%) retransplants for primary failure, 15 (20.0%) for graft rejection, and 29 (38.7%) for secondary failure, of which 6 (20.6%) were from other services. Previous studies showed that several factors may be associated with corneal transplant failures such as immune rejection, endothelial cell function of the transplanted bud, factors related to the surgical technique, and patient adherence to postoperative treatment, which includes immunosuppressant eyedrops^(23,24)^.

The limitations of this study implicate the inherent bias of retrospective studies, including incomplete data registry and loss of information. These are mentioned as possible drawbacks of the study. Nevertheless, the research allowed the extraction of the profile of patients who underwent keratoplasty in a reference eye center in Northeastern Brazil, which may guide future public health measures aimed at preventing diseases and reducing the necessity of corneal transplants.

The epidemiological profile of patients who underwent corneal transplantation in Pernambuco showed a similarity between the sexes and a predominance in the economically active population. In addition, the main indications for corneal transplantation were infectious keratitis, keratoconus, and post-transplant failure. These indications are preventable and treatable. Therefore, preventive measures and increased accessibility to health care are important public health strategies to reduce the social and economic impacts of corneal diseases in developing countries.
